# Requirements for Supporting Diagnostic Equipment of Respiration Process in Humans

**DOI:** 10.3390/s21103479

**Published:** 2021-05-17

**Authors:** Szymon Nitkiewicz, Robert Barański, Marek Galewski, Hanna Zajączkiewicz, Andrzej Kukwa, Andrzej Zając, Stanisław Ejdys, Piotr Artiemjew

**Affiliations:** 1 Faculty of Technical Sciences, University of Warmia and Mazury, 10-710 Olsztyn, Poland; 2 School of Medicine, Collegium Medicum, University of Warmia and Mazury, 10-082 Olsztyn, Poland; hanna.zajaczkiewicz@uwm.edu.pl (H.Z.); andrzej.kukwa@uwm.edu.pl (A.K.); stanislaw.ejdys@konin.edu.pl (S.E.); 3 Faculty of Mechanical Engineering and Robotics, AGH University of Science and Technology, 30-059 Kraków, Poland; robert.baranski@agh.edu.pl; 4 Faculty of Mechanical Engineering and Ship Technology, Gdansk University of Technology, 80-233 Gdansk, Poland; margalew@pg.edu.pl; 5 Institute of Optoelectronics, Military University of Technology, 00-908 Warsaw, Poland; andrzej.zajac@wat.edu.pl; 6 Faculty of Economics and Technical Sciences, State University of Applied Sciences, 62-510 Konin, Poland; 7 Faculty of Mathematics and Computer Sciences, University of Warmia and Mazury, 10-710 Olsztyn, Poland; artem@matman.uwm.edu.pl

**Keywords:** respiration, diagnostic equipment, physiologic monitoring, data acquisition, sleep apnea syndrome, sleep-disordered breathing

## Abstract

There is abundant worldwide research conducted on the subject of the methods of human respiration process examination. However, many of these studies describe methods and present the results while often lacking insight into the hardware and software aspects of the devices used during the research. This paper’s goal is to present new equipment for assessing the parameters of human respiration, which can be easily adopted for daily diagnosis. This work deals with the issue of developing the correct method of obtaining measurement data. The requirements of the acquisition parameters are clearly pointed out and examples of the medical applications of the described device are shown. Statistical analysis of acquired signals proving its usability is also presented. In the examples of selected diseases of the Upper Respiratory Tract (URT), the advantages of the developed apparatus for supporting the diagnosis of URT patency have been proven.

## 1. Introduction

**Figure 1:**
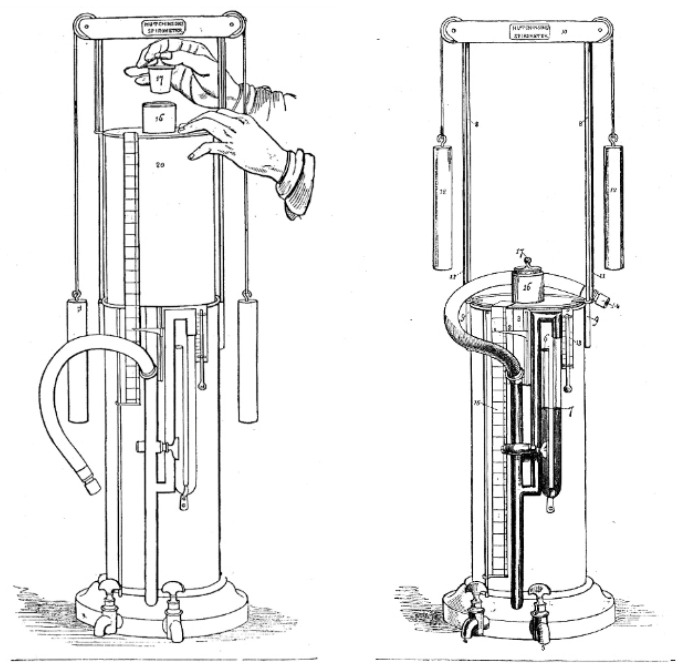
Drawing of Hutchinson’s spirometry tester [1,3,4].

Respiratory test instrumentation has improved significantly in recent years. The genesis of respiratory measurements is dated to the 19th century when, in 1846, the first trials on Vital Capacity (VC) were carried out, performed by Sir John Hutchinson [1]. However, it was not possible to take precise measurements with the equipment he developed, as shown in Figure 1.

The most popular functional test is the spirometry test and, particularly, forced expiration. Individual desired volumes are obtained on the basis of the determined, directly measured flow Q (t) (l/s), by using an electronic integrator according to the formula (1). The essence of this test is to measure the flow, Q (t), and the volume, V (t), during inhalation and exhalation. However, due to the complexity of this procedure, there are many incorrect and uncertain results in such medical trials [2]. Despite several disadvantages, spirometry remains the primary test of evaluation of human URT.

V (t) = ∫ Q (t) dt(1)

Another common test is plethysmography, which is carried out in a sealed cabin. This test measures the pressure changes inside the cabin, which are caused by the change in the volume of the patient’s chest. One of the first tests was performed by the Dutch biologist Jan Swammerdam in the year 1667. Nowadays, the examined person breathes through a mouthpiece, which allows the recording of the air stream *Q* (*t*). Plethysmography is a test which allows the assessment of the total air volume in the lungs by Total Lung Capacity (TLC). It also makes it possible to determine the Residual Volume (RV). The volume of the air in the patient’s lungs is calculated on the basis of pressure changes in the cabin and its known volume, based on Poisson’s and Boyle-Mariott’s laws [5,6,7]. The other way to calculate the RV is by using the gas dilution technique. These volumes cannot be determined using a classical spirometry test. With the use of a spirometer, it is possible to measure the exhaled volume of air, the residual one cannot be determined in such a trial, which is described in detail by Joseph Feher [8] and Frank Powel et al. [9].

Development of the measurement methodology led to the appearance of new methods based not only on new sensors but mostly in the way the measurements were performed. Interrupter Technique is a technique for measuring airway resistance. This method was proposed by von Neergaard and Wirtz in 1927 [10,11,12]. It is a minimally invasive method and its implementation is quick and simple in clinical applications. Additionally, what is important is that this procedure does not require special cooperation from the patient, as in the case of, for example, spirometry. This is important, for example, for the easy testing of children and infants [13]. The interrupter technique assumes that during normal breathing, in short-term and sudden airway closure, the oral pressure equalizes to the alveolar pressure. The time of interruption of the flow varies from a dozen ms to about 100 ms, depending on the type and structure of the airflow-closing element [14]. In this technique, resistance is calculated as the ratio of the change in pressure in the mouth. Calculations are made by linear regression and the flow is measured just before the occlusion.

Recently, there has been an increase of interest in measuring human respiration monitoring, but most of the concern is focused on breathing rate, not the quantitative measurements of inhaled air [15,16,17]. Unlike in this article, published studies in this field do not separate the flow of air flowing into three channels; two nasal and one oral [18,19,20,21,22,23]. This article is dedicated to describing and proposing new measuring equipment which combines the ease of use of the Interrupter Technique with spirometry tests. The great benefit of the proposed device is its accuracy and measurement repeatability [24]. In this paper, we would like to present both the basic medical background of our research and the requirements that were set while building a new device, which would be helpful in the rapid assessment of URT patency.

## 2. Medical Background

**Figure 2:**
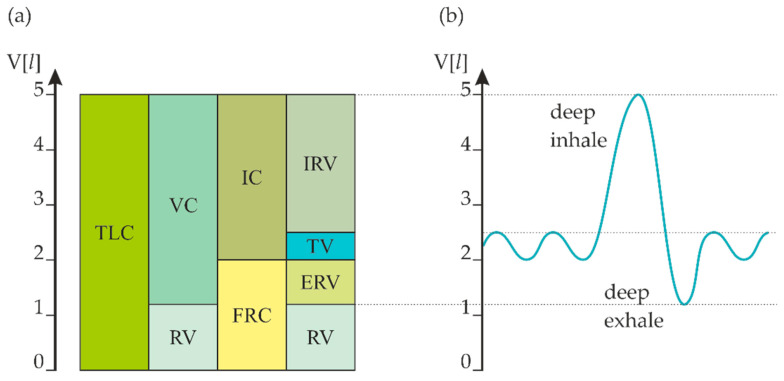
(**a**) The division of volume and respiratory capacity of a healthy person, (**b**) the curve of changes in volume during breathing (based on [25,26]).

The breathing movements, caused by the muscles, move and hold the air in the airways and lungs. A clinical representation of human retained air is shown in Figure 2 [25,26,27,28], with the following acronyms: Total Lung Capacity (TLC), Vital Capacity (VC), Residual Volume (RV), Inspiratory Capacity (IC), Functional Residual Capacity (FRC), Inspiratory Reserve Volume (IRV), Tidal Volume (TV), Expiratory Reserve Volume (ERV). Relationships between the quantities marked in Figure 2a can be calculated based on ‘*A Report of the ACCP-A TS Joint Committee on Pulmonary Nomenclature*’ and are presented in Equations (2)–(7) [29]:

TLC = VC + RV(2)

TLC = IC + FRC(3)

TLC = IRV + TV + ERV + RV(4)

IC = IRV + TV(5)

FRC = ERV + RV(6)

VC = IRV + TV + ERV(7)

The physiological values of respiration parameters depend on several factors. One of the major factors influencing these traits is gender and age. When determining breathing parameters for children, a detailed breakdown by age is distinguished in groups of newborns, toddlers, one-year-olds, two-year-olds and older [30]. Selected data for adults and children over 6 years of age are presented in Table 1 [2,26,28,30,31].

Disorders of the breathing process may also concern parameters such as [32]:

- time—breathing rate (slow, fast, irregular breathing);
- the amount of air taken from the external environment (deep, shallow breathing);
- disturbances in chest movement (unilateral breathing);
- changes in the respiratory tract (in the case of men, the dominant path is the abdominal path—associated with the movement of the diaphragm, and, in the case of women, the thoracic path).

The acceleration of breathing—tachypnoea—can be related to either the respiratory system (pneumonia) or cancer. Furthermore, changes in breathing parameters in relation to the volume of air taken during each breath may accompany diseases of a diabetic nature and diseases of the respiratory system, such as Chronic Obstructive Pulmonary Disease (COPD) [33].

A breathing disorder consisting of the occurrence of apnea lasting several seconds, after which the respiratory action returns, reveals itself in the form of an increase in the amplitude of the volume and respiratory rate to the maximum value and then slows down until the next apnea episode. This disorder is called Cheyne-Stokes respiration and is associated with diseases of the central nervous system. Acceleration above 40 times per minute, or slowing down of breathing below 8 times per minute, in the case of an adult, is a very serious symptom because it can lead to hyperventilation or hypoxia [31,34,35,36,37,38,39].

## 3. Data Acquisition Requirements

**Figure 3:**
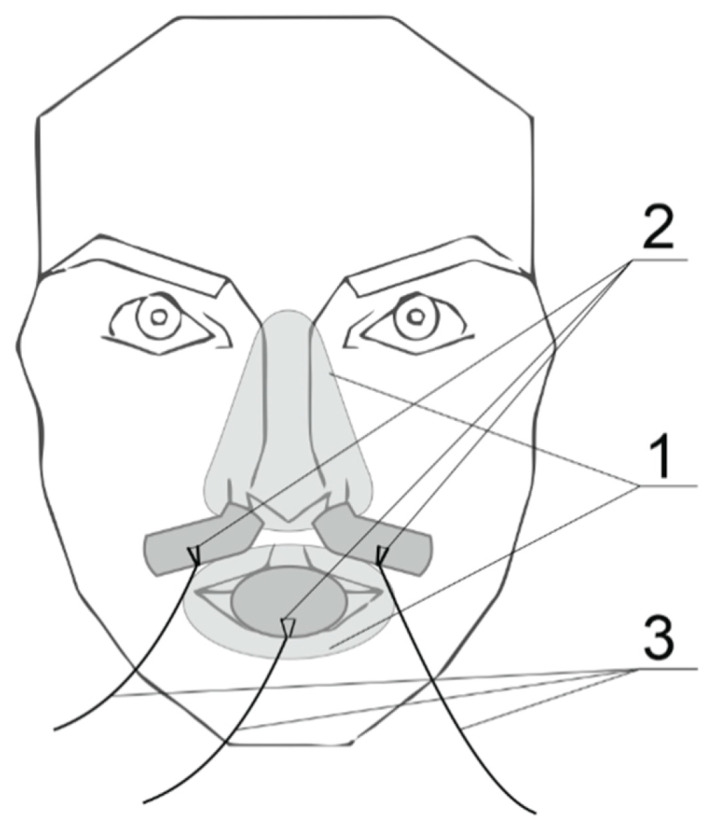
Concept of the proposed device where 1 = holding elements for sensors, 2 = hot-wire sensors, 3 = data cables.

In most measurement procedures, it is not possible to observe the movement of a single molecule that makes up the entire liquid or gas, the flow of which is observed. The exceptions are microscopic measurements of Brownian motion or measurements of the movement of isotope-labeled particles, as well as measurements with isotope contrast agents; absorption (X-ray, CT, MRI) or thermal (such as Transit Time Flow Measurement, especially in cardiovascular surgery) [40,41]. Thus, the measurement of fluid flow, particularly airflow, is considered macroscopically as the overall movement of the fluid; the stream of the measured medium. Pressure, volume or velocity as physical quantities allow the motion and state of a fluid to be described in terms of the conservation of energy and momentum [38]. In the introduction, some respiratory disorders were described as well as the known and widely-used measurement methods by which we can detect them. However, these methods are not sufficient for determining the respiration patency over extended temporal recordings. Long-term analysis is required for the evaluation of breathing disorders like apnea or displacing of the nasal valve. For the correct assessment of these syndromes, a reliable source of data acquisition is required. Therefore, the assessment of URT patency using the recently developed device was proposed [24,42]. Its great advantage is the continuous 3 channel measurement system for all human breathing openings, see Figure 3.

During the development of the device, some essential assumptions, like small size, mobility or low power consumption of the device, were made.

Various sensor types were considered but, finally, hot-wire anemometers were selected due to their perfect metrological parameters that meet the assumptions of the system built. The main advantages of thermoanemometric sensors are the possibility of sampling the signal with a rate of up to about 100 kHz and measuring the speed of the flowing medium of about 30 m/s using a measuring element (wire) with a length of 3 mm and a diameter of 5 µm [34,35,36,37]. It must be noted that these parameters greatly exceed the needs of the proposed system and the selection of the actual sampling rate is described in Section 3. Another advantage of hot-wire anemometers is the miniature size of these sensors that practically do not disturb the air stream flowing through it. They also have low power consumption.

**Figure 4:**
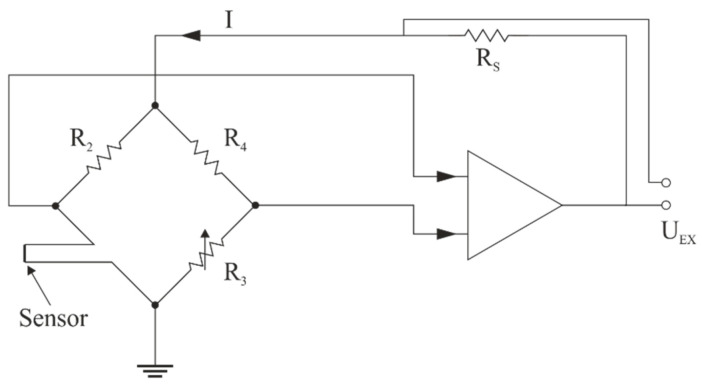
Diagram of the anemometer in a CTA configuration (based on [43]).

**Figure 5:**
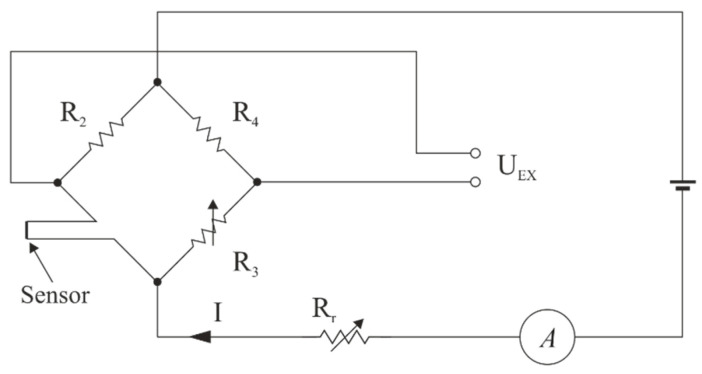
Diagram of the anemometer in a CCA configuration (based on [43]).

There are two well-known types of thermo-anemometers; the Constant Temperature Anemometer, CTA (T_w_ = const.), where the temperature of the wire is kept constant, regardless of the speed of the flowing fluid; and the Constant Current Anemometer, CCA (I_w_ = const.), the principle of which is to maintain a constant current, unchanged during the measurement of the medium flow. Schematic diagrams of bridge systems for both types of thermo-anemometers are shown in Figure 4 and Figure 5.

In the CCA configuration, there is a danger of burning the wire/fiber if the cooling airflow is insufficient. Likewise, if the flow is too high, the wire does not heat up enough to provide satisfactory accuracy of the measurements. For these reasons, most thermo-anemometers work in a constant temperature configuration [43,44,45,46]. Considering the properties of individual thermo-anemometer configurations, a CTA was selected for use in the described device. Detailed mathematical models of thermoanemometric flowmeters can be found in the work of Korobiichuk et al. [47]. One of the properties of this solution is, as mentioned earlier, the protection against overheating/burning of the sensor wire in the absence of airflow. This is possible, for example, when performing measurements on a patient suffering from sleep apnea.

**Figure 6:**
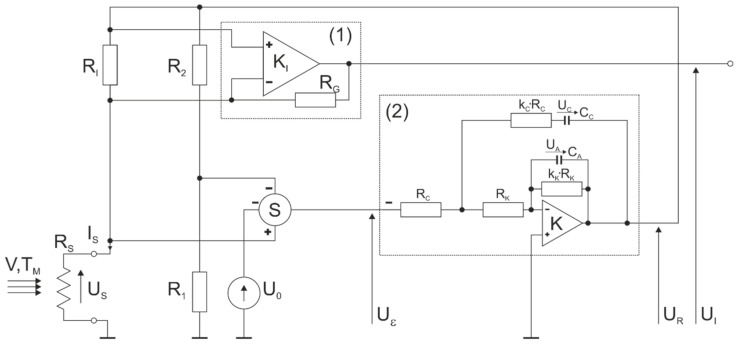
Constant temperature thermo-anemometer in a bridge configuration [48]. Where *R_S_* = measuring element (thermo-anemometer), *U_S_* = sensor voltage, *I_S_* = sensor current, *V* = speed of the medium, *T_M_* = medium temperature, *R*_1_, *R*_2_, *R_I_* = bridge-forming resistors, *R_G_* = gain resistance, *K_I_* = differential amplifier, *S* = adder, *U_R_* = bridge supply voltage, *U_0_* = offset voltage, *U_ε_* = error voltage, and *U_i_* = output voltage.

The developed measuring system makes it possible to determine both the duration of the individual phases of the breath and to quantify it by computing the volume of exhaled air. These values can be determined independently for both nostrils and mouth. The diagram of a single measurement path of a constant temperature thermo-anemometer is shown in Figure 6 [48]. As mentioned earlier, the device consists of a three-channel measurement.

The output signal from the described system is the voltage U_I_ proportional to the current I_S_ flowing through the sensor according to the relation (8):

U_I_ = K_I_ R_I_ I_S_ (8)

where U_I_ = output voltage (V), K_I_ = signal amplification, R_I_ = resistor resistance (Ω), I_S_ = sensor current of R_S_ [A].

For this purpose, a reliable Data Acquisition (DAQ) unit must be considered. Various types of DAQ equipment, starting from low-cost Arduino boards up to highly sophisticated units such as NI (National Instruments, Austin, TX, USA) Multifunction I/O devices [49,50,51]. The choice should be made taking into consideration many aspects like expected system accuracy, reliable sampling and its timing, hardware and software features, development time, available budget and others. In the described device the NI-6002 USB Data Acquisition Card was used.

NI-6002 DAQ card contains 4 Differential or 8 Single-ended Analog Inputs, 16-Bit Analog to Digital resolution converter and offers sampling rates up to 50 kSamples/s [52]. Its measurement capabilities fulfill the requirements to a sufficient degree [24]. The sampling rate of the NI-6002 DAQ card is good enough to measure the breathing rate and changes over time, which are explained in the next section of this paper.

### Methodology of Measurement

**Figure 7:**
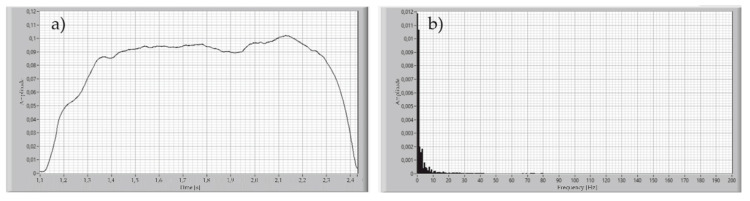
Time plot of the single inhale of the healthy participant (**a**), and Fourier transform of this signal (**b**).

**Figure 8:**
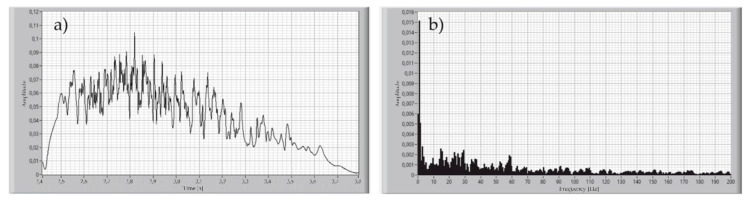
Time plot of the single exhale of the healthy participant (**a**), and Fourier transform of this signal (**b**).

For determining the signal acquisition parameters, the breath of healthy participants was recorded with the proposed device. The sampling rate of the measured signal was set to be 1000 Samples/s. The significant variability of the archived signal resulting from the Fourier transform was 8 Hz for inhale and more than 180 Hz to exhale, as shown in Figure 7 and Figure 8.

**Figure 9:**
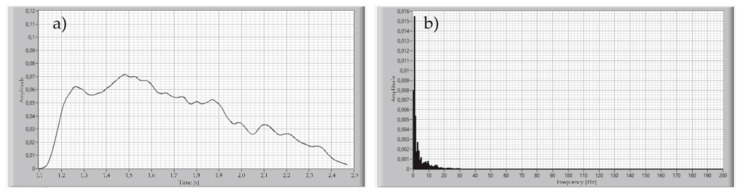
Time plot of the single exhale of the healthy participant after applying a 10 Hz low-pass filter (**a**), Fourier transform of this signal (**b**).

This means that the sampling frequency for this signal must be set to at least 360 Hz, according to the Nyquist theorem. It was assumed that the high components of the signal spectrum were caused by the humidity of the exhaled air, due to the low value of the heat capacity of the measuring element from which the sensor is made. On the other hand, the calculations conducted for the estimation of the breathing rate, and volume of the single inhale or exhale, do not require such a good sampling rate. For that reason, a Butterworth 2nd order, low-pass 10 Hz filter was applied during the measurements of the signal of exhaled air. The result of this operation is shown in Figure 9.

## 4. Verification of Measurements

**Figure 10:**
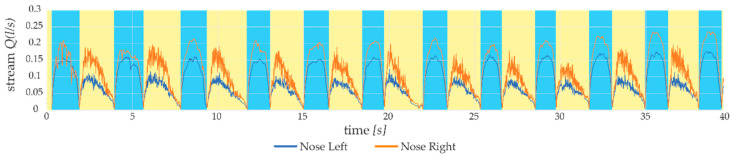
Separated left and right nostril breathing measurements.

To evaluate the measurement method proposed in the article, a number of tests were carried out to verify the metrological features of the developed device. Measurements of the airflow within the sensors, presented in Figure 10, show the recorded examples of values during breathing for the right and left nostrils. The airflow *Q* (*l*/*s*) is marked on the vertical axis and the elapsed time of the measurement is shown on the horizontal axis. The sampling frequency of the presented trial was set to 2 kHz. As it can be seen, the shape of the graph showing the flow rate of air differs significantly during exhalation and the inspiratory phase. The graph shows the recorded stream for the ‘Nose Left’ sensor with the blue line and the recorded stream for the ‘Nose Right’ sensor with the orange line. Areas marked with a blue background are identified phases of inspiration, while areas marked with a yellow background are recognized phases of exhalation.

The air exhaled by humans has nearly 95% humidity and a temperature of about 34.8 °C [53,54,55,56,57]. The relative humidity of the exhaled air depends very much on the temperature. The air in the lungs is saturated with water at 37 °C, i.e., has a vapor pressure of about 47 mmHg or water content of about 44 mg/L. As stated earlier, this leads to significant fluctuations of the measured signal. This effect can be eliminated in the applied calculation procedure, assuming the obvious condition of gas stream continuity during inhalation and exhalation, they must be closely related.

**Figure 11:**
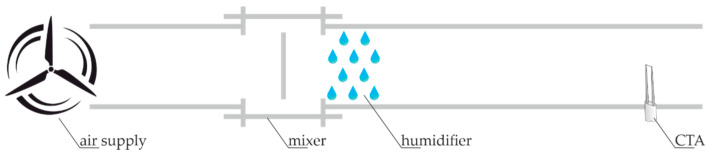
Diagram of the constructed test measurement track.

To verify this assumption, a control system was built that simulates breathing with dry air (inhale) and moist (exhale). The constructed system was tested in terms of repeatability of measurements both in a dry environment and after moistening the flowing air. The system consists of a fan (marked as air supply in Figure 11) with adjustable efficiency of the flowing air, a mixer and a humidifier, for the case of moist air measurements. At the end of the measurement path, there is a constant temperature thermo-anemometer (marked CTA) selected for use in the discussed device. In the second phase of the tests, the temperature of the air flowing through the test track (Figure 11) measured near the sensor was 19 °C and the humidity was 94%.

**Figure 12:**
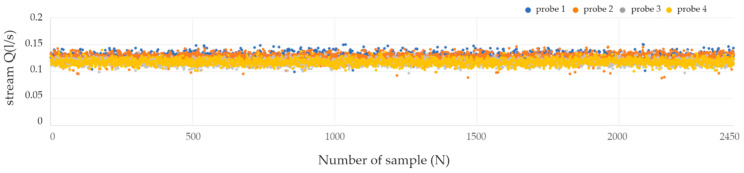
Chart of acquired air stream measurements for the fan supplied with 12V DC voltage.

In the chart (Figure 12), on the vertical axis, the values of the air stream flowing through the sensor Q(l/s) are marked. The voltages recorded by the sensors correspond to the air stream, by solving the transition function acquired when calibrating the sensor by the manufacturer. In the proposed evaluation system, the fan is supplied with *DC 12V* (as a representative example). All the sensors were tested: two used as nasal, one for oral paths and, additionally, one spare sensor. The fan supplied with DC 12V voltage creates an airflow in the measuring channel similar to moderate human breathing. To verify the measurements with the use of individual sensors, a continuous measurement lasting for 2 min was adopted for each tested sensor. The sampling rate of the signal from the sensors was 200 samples per second.

The analytical values of the conducted trials are presented below in Table 2.

**Figure 13:**
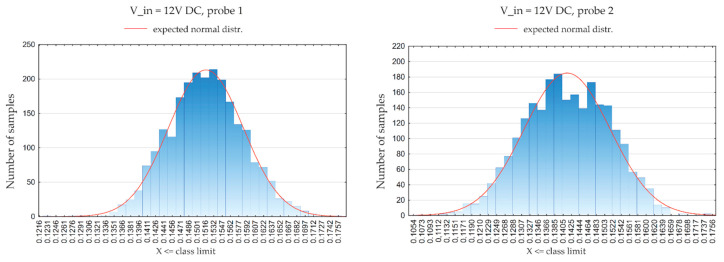
Chart of distribution of the measured values of the *probe 1* and *probe 2* sensors with a supplying voltage of *12 V DC.*

The measured values of the stream presented in the chart (Figure 13) show the distribution of measurement data for one selected sensor—probe 2. To test whether the distribution of samples recorded during a single measurement is consistent with the normal distribution (which is necessary for further tests) the Lilliefors test was used [58]. The Lilliefors test is based on the Kolmogorov-Smirnov test (K-S test), which could not be used due to the large number of samples tested. For the K-S test, it is advisable that the cases were less than 2000, otherwise, it may lead to erroneous results. Additionally, this test requires knowledge of the mean and standard deviation of the population. In the analyzed situation, the above-mentioned parameters are not known. In the absence of this information, the K-S test with the Lilliefors correction (Lilliefors test) is applied. Limiting the number of cases (measurement points) to the number corresponding to the K-S test did not affect the test result, or the determination of the normality of the distribution of samples, additionally the K-S test also requires the feature to be continuous.

The results of the verification tests of the normality of distribution are summarized in Table 3. Lilliefors’ test value (*p*) below *0.05* indicates rejection of the null hypothesis (H_0_) assuming normal distribution. These values are marked in red in the table below. During the experiments, an analysis of changes in atmospheric humidity during breathing was conducted. As for the tests carried out with dry air, the stream Q (l/s) of the air flowing through the sensor was measured and marked on the graph on the vertical axis. The horizontal axis shows the sample number (with sampling 200 samples/s).

**Figure 14:**
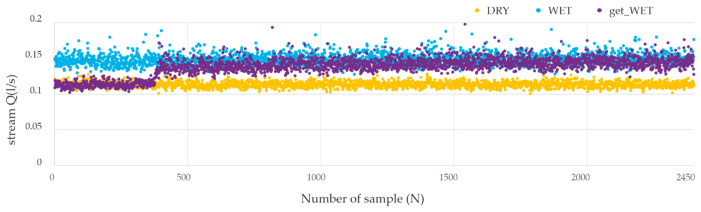
Chart of acquired air stream measurements for the fan supplied with 12V DC voltage for dry, wet and ‘getting wet’ air conditions.

The graph (Figure 14) presents the course of the values for dry air (room humidity, according to the norm [59]), the change of the stream value during humidification, as well as the recorded measurements of the humidified air. The temperature of the air flowing through the test measurement system, measured near the sensor, was 19 °C and the humidity was *94%*. Analytical data for the described measurement are also presented below in Table 4. The classes of humidity conditions, in tables are: DRY = 50%, getting WET = 50%→94%, WET = 94% of humidity.

Furthermore, as for the measurements from the first part of the tests, an analysis was carried out to check whether the distribution of samples recorded during a single measurement is consistent with normal distribution. The same statistical method was used, i.e., the Lilliefors test [58]. The values obtained as a result of the analysis are presented in Table 5.

Any statistical method of data processing should include an analysis of the uncertainty of their evaluation. In accordance with the requirements, one type of evaluation with direct measurements is a type A uncertainty. It concerns the statistical analysis of the set {*x_i_*} of independent measurements repeated *n* times. There are two benefits of repeating a measurement with a random error: an increase in the accuracy of the measurement by calculating the mean value, and the possibility to assess the uncertainty of this measurement [60,61,62].

The error analysis presented in Table 6 was carried out for the probe 2 sensor for the same value of fan speed for the ‘DRY’ and ‘WET’ humidity airflow conditions.

The value of the estimator of the standard deviation of the average of *ESD_A_* = 2 − 10^−4^, which indicates a negligible impact on the result of a single measurement. Additionally, by applying the obtained error to the values measured during the tests, it can be concluded that, with the average length of inhalation/exhalation lasting about 2 s, the error resulting from the measurement of a particular breath will be at the level of individual milliliters, which does not affect the quality of the assessment of airway patency. The very good stability of the laboratory measurements should also be noted. In each of the test measurements carried out, over 95% of the measured values were within the range of the mean value ±3 standard deviations. The percentage of measurements falling within the so-called 6σ (mean ±3σ) is presented in Table 7.

As can be seen from the data and conducted analysis, the device and performed measurements could have great potential in supporting the diagnosis of URT disorders. Repeatability and accuracy of the assembled device are at a high level of trust. In the paper, we recall only a part of the verification tests that were performed to establish the reliability of the proposed device. After completing the confirmation of the device properties, some medical supporting attempts were conducted. Appropriate Bioethical Commission approval was issued in April 2017 (see Acknowledgments). Overall, more than 100 patients were tested with the use of the described device. As the device is suitable for trials with children, a large part of the tests are measurements of breathing difficulties caused by an enlarged third tonsil [63]. In the case of tests conducted in adult patients, the disorders ranged from deviated septum nasal to turbinate hypertrophy and others.

## 5. Medical Application

In this section, a few medical applications of the described device are shown, starting from the common disorder sleep apnea. Sleep breathing problems are a heterogeneous group of issues that include obstructive sleep apnea syndrome. The main causes of breathing disorders include craniofacial developmental disorders and upper airway anomalies. A significant factor that predisposes to or intensifies sleep apnea symptoms is obesity.

Undiagnosed obstructive sleep apnea syndrome (OSAS) contributes to the development of civilization diseases such as hypertension and diabetes. Consequently, it may be the cause of cardiovascular diseases [64,65,66].

**Figure 15:**
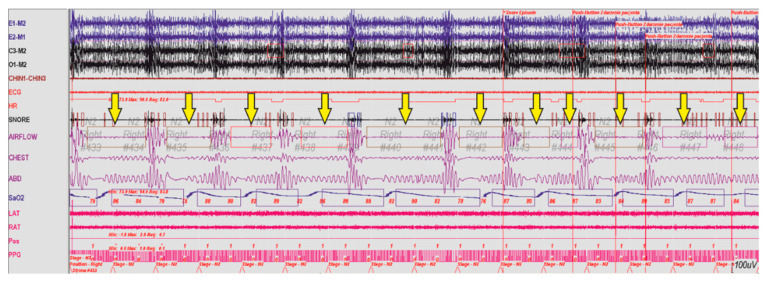
Fragment of a polysomnographic examination of a patient with breathing disorders during sleep, the arrow marks episodes of apnea.

By detecting the risk of OSAS, we can indirectly prevent its development. Currently, the diagnostics of patients with sleep breathing disorders consists of conducting otolaryngologic or videoscopic examinations, as well as imaging studies (CT) and so on. A polysomnographic examination, colloquially also known as ‘sleep study’ consists of many procedures such as Electrocardiography (ECG), Electrooculography (EOG), Electroencephalography (EEG), Surface Electromyography (SEMG), Pulse Oximetry and oxygen saturation (SpO_2_). One of the elements of this study is monitoring patient’s respiratory activity. A fragment of a recorded polysomnographic examination is presented in Figure 15.

**Figure 16:**
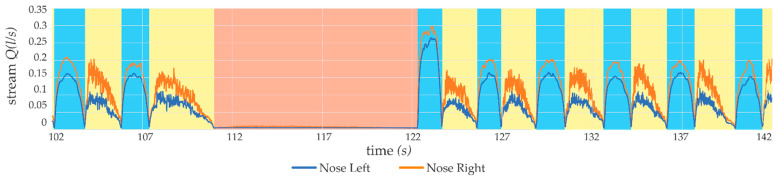
Fragment of a recorded breathing action with apnea episode marked in red.

The information provided by the analysis of the presented case indicates the occurrence of numerous episodes of sleep apnea. As respiration stopped the blood oxygenation (SpO_2_) also decreased. The described device has a feature to continuously monitor the breathing action and to warn when such a situation occurs. Part of the recorded breath action can be seen in Figure 16, where two nostrils breathing were monitored. The apnea episode is marked with the red color, breath in and out, respectively, with blue and yellow.

According to practical experience, the sampling frequency was raised to 1 kHz, which is much above the minimum requirements. This was done for the purpose of further signal analysis in future works.

**Figure 17:**
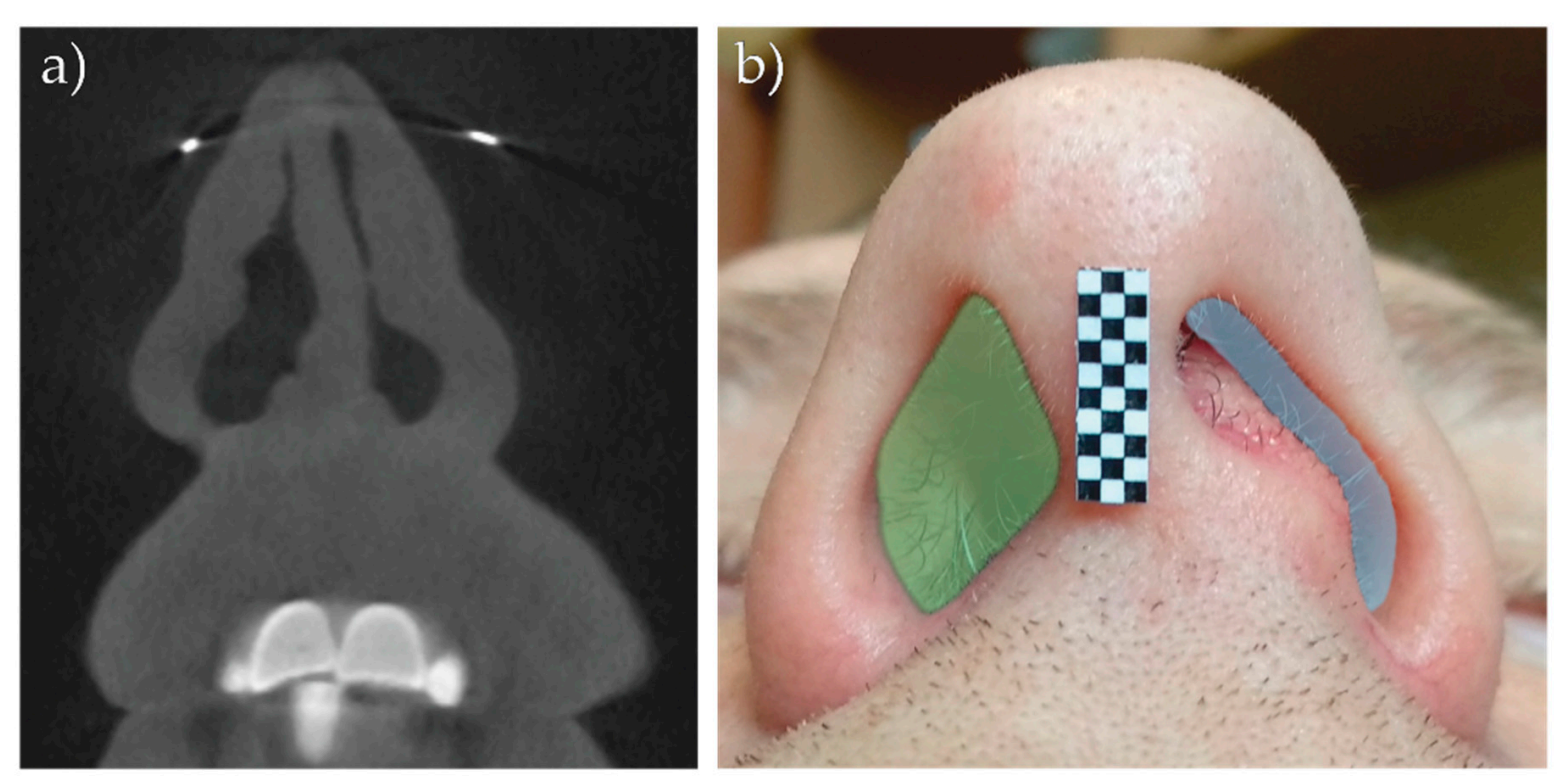
(**a**) Computed Tomography scan of a patient with deviated septum nasal; (**b**) photo with marked nostrils surface (light blue 25 mm^2^, light green 69 mm^2^, marker with 1 × 1 mm measure reference).

The following case was performed with the participant suffering from URP disease and was conducted in the Otorhinolaryngology Clinic of University Clinical Hospital in Olsztyn, Poland. The patients hospitalized in the clinic, depending on disorders, have additional medical imagining examinations conducted, such as Computed Tomography (CT), Cone Beam Computed Tomography (CBCT) or Magnetic Resonance Imaging (MRI). An example of a CT scan of one of the cases (deviated septum nasal) is presented in Figure 17. Moreover, a photo and measurements of the patient’s nose were taken. In both pictures, we can see a big asymmetry in the nasal area. Such a disorder, caused by obstructed and nonsymmetrical airflow through the nostrils, can lead to apnea, especially in sleep.

**Figure 18:**
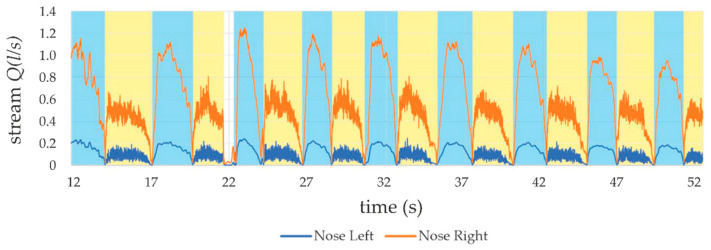
Asymmetry in deep breathing (areas: blue: inhale; yellow: exhale).

Nostrils, as the end of the respiratory tract, have a great impact in limiting human breathing ability. Measured surfaces of the nostrils, left and right, respectively, 25 mm^2^ and 69 mm^2^, indicating an obstruction of the patient’s air exchange. This was also proven with measurements performed with the described device. Great asymmetry in the air stream can be observed in Figure 18. These measurements were taken while the patient was breathing deeply. 

**Figure 19:**
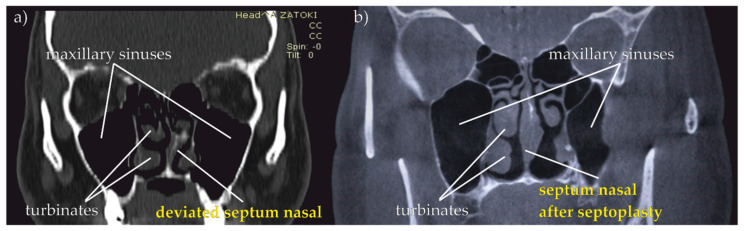
Computed Tomography scan of a patient with deviated septum nasal (**a**) before surgery; (**b**) after surgical intervention.

In cases of anatomical anomalies such as tonsillar hypertrophy, nasal polyps, septal tilt, hypertrophy of the palatine uvula and flaccid palate, the intervention of an otorhinolaryngologist is necessary. The proposed device allows us to objectively assess the problem of breathing and airway obliteration at different heights. Then it allows us to qualify the patient for otolaryngological procedures such as tonsillectomy, uvulopalatoplasty, polypectomy or septoplasty, which eliminate the causes of upper airway obstruction. The effect of surgical intervention can be seen in Figure 19.

**Figure 20:**
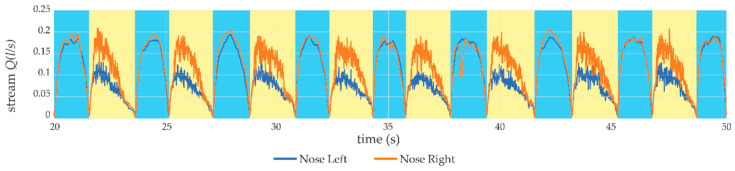
Measurements taken after surgery proving an improvement in the patient’s breathing.

In Figure 19 an effect of the nasal septum surgery in the form of opening the URT can be seen. In the subjective assessment of the patient, after a few weeks, he began to breathe freely without a feeling of obstruction in his nose. This feeling was confirmed by repeating the measurements with the use of the proposed device, which can be seen in Figure 20.

The device helps in the postoperative assessment of the effect of airflow through the upper airways at different heights, allowing a comparison of breathing quality before and after surgery and aiding the determination of the next medical steps or completion of the treatment.

## 6. Conclusions

It is possible to develop a mobile diagnostic kit allowing for the objective analysis of selected Upper Respiratory Tract disease states by selecting and developing a dedicated measurement method, as well as significantly improving and objectifying the process of diagnosing respiratory disorders. Until now, spirometry has been the main examination for impaired airway patency, now it is recognized as the so-called ‘gold standard’ for assessing the patency of human URT. The above-mentioned method has great diagnostic value but does not provide answers to several important aspects of respiratory disturbances, such as the symmetry of airflow through the nose (i.e., a detailed patency test). Separation of the measurement channels into 3 paths, one oral and two nasal, allows for independent, simultaneous measurement of breathing in each of the mentioned channels.

Due to the innovative nature of the measurements and tests proposed in the article, the assessment of the patient’s airway patency performed with the use of the discussed device was confirmed by additional imaging tests, such as MRI or CBCT. The proposed method is not time-consuming, the measurement time during a single examination is about 5 min for an outpatient measurement (ambulatory), up to 6–8 h (that is the same as during the procedures performed so far with the polysomnography test) for measurements during sleep.

Studies conducted (with the use of the described device) on volunteers hospitalized at the University Clinical Hospital in Olsztyn, Poland, clearly indicate the need to assess the patient’s condition in the scope described in the presented article. The diagnostic possibilities with the use of the aforementioned device accelerate diagnostics in the area of Upper Respiratory Tract diseases, which specialists in the field of laryngology meet daily. Our device, along with the patient’s blood saturation test and other tests, will benefit in the diagnosis of obese people and people with diabetes. We believe that the separation of measurement channels (nasal and oral) during trials may improve the diagnosis and facilitate the work of specialists in the field of medicine.

Properties of the device presented in this paper, after it obtains medical certification, could be a valuable supporting tool for determining URT disorders. It is not easy to meet the requirements of the certification process in the field of medical devices, especially in the area of compliance with EU and other international standards and regulations. This manuscript aims to explain that further studies will be needed in order to test the device and its applications.

## 7. Patents

The device described in this paper has been also a subject of patent pending in November 2016, with no. P.419511. In July 2020, the Polish Patent Office granted a patent for an invention ‘Control equipment for monitoring patency of respiratory tract’, No. Pat.236745.

## Figures and Tables

**Table 1:** Physiological values of human respiration parameters.

Trait	Value for an Adult	Value for a Child
Respiratory rate (f)	12–20 /min	14–24 /min
Total Lung Capacity (TLC)	3.2–6,0 L	depends on age
Tidal Volume (TV)	0.5 L or 7–8 mL/kg bw *	7–8 mL/kg bw *
Vital capacity (VC)	4.5–5.0 L	depends on age
Functional Residual Capacity (FRC)	2.5–3.0 L	depends on age
Residual volume (RV)	1.2 L	0.5–0,8 L
Forced expiratory volume in 1st sec. (FEV1)	75% VC	75% VC

* bw—body weight.

**Table 2:** Values for conducted trials.

Statistical Feature	*Probe 1*	*Probe 2*	*Probe 3*	*Probe 4*
Mean *Q* (*l*/*s*)	0.1276	0.1254	0.1168	0.1177
Median *Q* (*l*/*s*)	0.1275	0.1256	0.1167	0.1177
Standard deviation (*SD*) *Q* (*l*/*s*)	0.0065	0.0070	0.0052	0.0054
Min value *Q* (*l*/*s*)	0.0995	0.0880	0.0969	0.0991
Max value *Q* (*l*/*s*)	0.1505	0.1463	0.1379	0.1398
Number of samples (*N*)	2400	2400	2400	2400

**Table 3:** The results of the sample normality distribution test (Lilliefors test) for the supplying voltage of the fan V_in = 12V DC, probes 1–4.

Probe No.	Lilliefors Test Val.	*Normal Distr.*
1	*p* > 0.10	YES
2	*p* < 0.05	NO
3	*p* < 0.01	NO
4	*p* > 0.20	YES

**Table 4:** Values for conducted trials.

Statistical Feature	*DRY*	*Getting WET*	*WET*
Mean val. Q (l/s)	0.1208	0.1455	0.1561
Median Q (l/s)	0.1206	0.1476	0.1554
Standard deviation (SD) Q (l/s)	0.0039	0.0129	0.0079
Min value Q (l/s)	0.1080	0.1098	0.1352
Max value Q (l/s)	0.1367	0.2054	0.2188
Number of samples (N)	2400	2400	2400

**Table 5:** The results of the sample normality distribution test (Lilliefors test) for the supplying voltage of the fan V_in = 12V DC, for different humidity conditions.

Humidity Conditions	Lilliefors Test Val.	*Normal Distr.*
DRY	*p* < 0.01	NO
getting WET	*p* < 0.01	NO
WET	*p* < 0.01	NO

**Table 6:** The results of the error analysis for *probe 2.*

Humidity Conditions	*Mean Val.*	*ESD*	*ESD_A_*
DRY	0.1208	0.0039	0.0001
WET	0.1561	0.0079	0.0002

where ESD = estimator of the standard deviation, and ESD_A_ = estimator of the standard deviation for average.

**Table 7:** Percentage of the measurement resulting within the range of mean ± 3σ.

Fan Supplying Voltage	Probe 1	Probe 2	Probe 3	Probe 4
12V DC	98.6%	99.4%	99.1%	99.5%

## Data Availability

Data available on request due to restrictions eg privacy or ethical.
